# S203. COMPENSATORY COGNITIVE APPROACHES TO IMPROVING FUNCTIONING IN PSYCHOSIS: SYSTEMATIC REVIEW AND META-ANALYSIS

**DOI:** 10.1093/schbul/sby018.990

**Published:** 2018-04-01

**Authors:** Kelly Allott, Kristi van-der-EL, Emma Parrish, Chris Bowie, Sean Kidd, Susan McGurk, Sarah Hetrick, Shayden Bryce, Matthew Hamilton, Eoin Killackey, Dawn Velligan

**Affiliations:** 1 Orygen, The National Centre of Excellence in Youth Mental Health; 2 Orygen, The National Centre of Excellence in Youth Mental Health, The University of Melbourne; 3 Harvard Medical School at Beth Israel Deaconess Medical Center; 4 Queen’s University; 5 University of Toronto; 6 Boston University; 7 University of Auckland and Centre for Youth Mental Health, University of Melbourne; 8 Monash University; 9 University of Texas Health Science Center at San Antonio, School of Medicine

## Abstract

**Background:**

Cognitive impairments in domains such as attention, memory, processing speed and executive functions are a central feature of psychotic disorders that have significant negative consequences for daily functioning, including activities of daily living, social and vocational roles. Compensatory approaches aim to minimise the impact of cognitive impairment on daily functioning through the use of aids or strategies to reduce cognitive load, in much the same way as glasses reduce the impact of vision impairment. The primary treatment target is real world community functioning and functional capacity, rather than cognition. There is now a need to synthesise the available evidence in this field so that treatment recommendations and future research directions can be better informed. A large body of research into compensatory approaches to cognition in psychosis exists, but this has never been comprehensively synthesised. The aim of this systematic review and meta-analysis is to examine the effects of compensatory approaches for cognitive deficits in psychotic disorders on i) functional outcomes and ii) other outcomes such as symptoms and quality of life.

**Methods:**

A systematic review and meta-analysis was conducted according to PRISMA guidelines. PsycINFO and MEDLINE electronic databases were searched from inception to October 2017 using multiple terms for ‘psychosis’, ‘cognition’ and ‘compensatory’. All papers retrieved from this search were double-screened and final inclusion/exclusion was determine by consensus. Data were double-extracted and risk of bias rated by two independent authors. Meta-analysis only included randomised-controlled trials. Standardised Mean Differences (SMD) were calculated to produce a single summary estimate using the random-effects model with 95% Confidence Intervals using Comprehensive Meta-Analysis (CMA) software. When means or standard deviations were not reported in the original articles, SMDs were calculated from data provided by the study authors.

**Results:**

2192 articles were identified via electronic and manual searches. Forty-two papers describing 40 independent studies were included in the review: case studies (n=4), case series (n=2), uncontrolled single arm pilot studies (n=5), within-subjects designs (n=1), quasi-randomised trials (n=2), and randomised controlled trials (n=26). The types of compensatory interventions included environmental adaptation and supports, internal and external self-management strategies, and errorless learning. Compensatory interventions were associated with improvements in global functioning post intervention (N=1,449; SMD=0.506; 95%CI=0.347, 0.665; p<.001). Improvements in global symptoms (N=849; SMD=-0.297; 95%CI=-0.484, -0.111; p=.002) and positive symptoms (N=784; SMD=-0.227; 95%CI=-0.416, -0.038; p=.018) were also found. Compensatory interventions were not associated with improvements in negative symptoms (N=736; SMD=-0.162; 95%CI=-0.382, 0.058; p=.150). The heterogeneity of findings was low.

**Discussion:**

Compensatory approaches are effective for improving functioning in psychosis, with a medium effect size. General symptoms and positive symptoms appear to benefit from compensatory approaches, but compensatory approaches are not effective for improving negative symptoms. Future analyses will examine the durability of effects, effects of study quality and moderating factors such as pure vs. partially compensatory, treatment intensity/length, mode of delivery (group vs. individual), baseline functioning level and age of participants.

